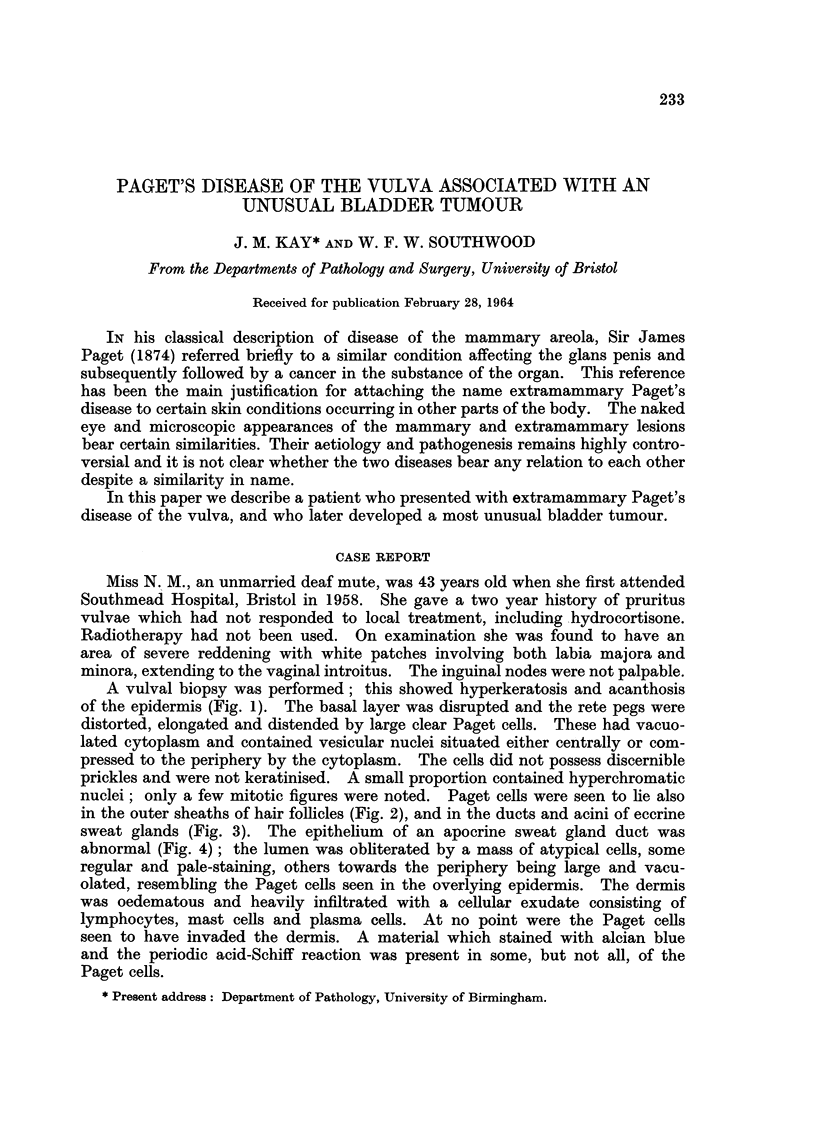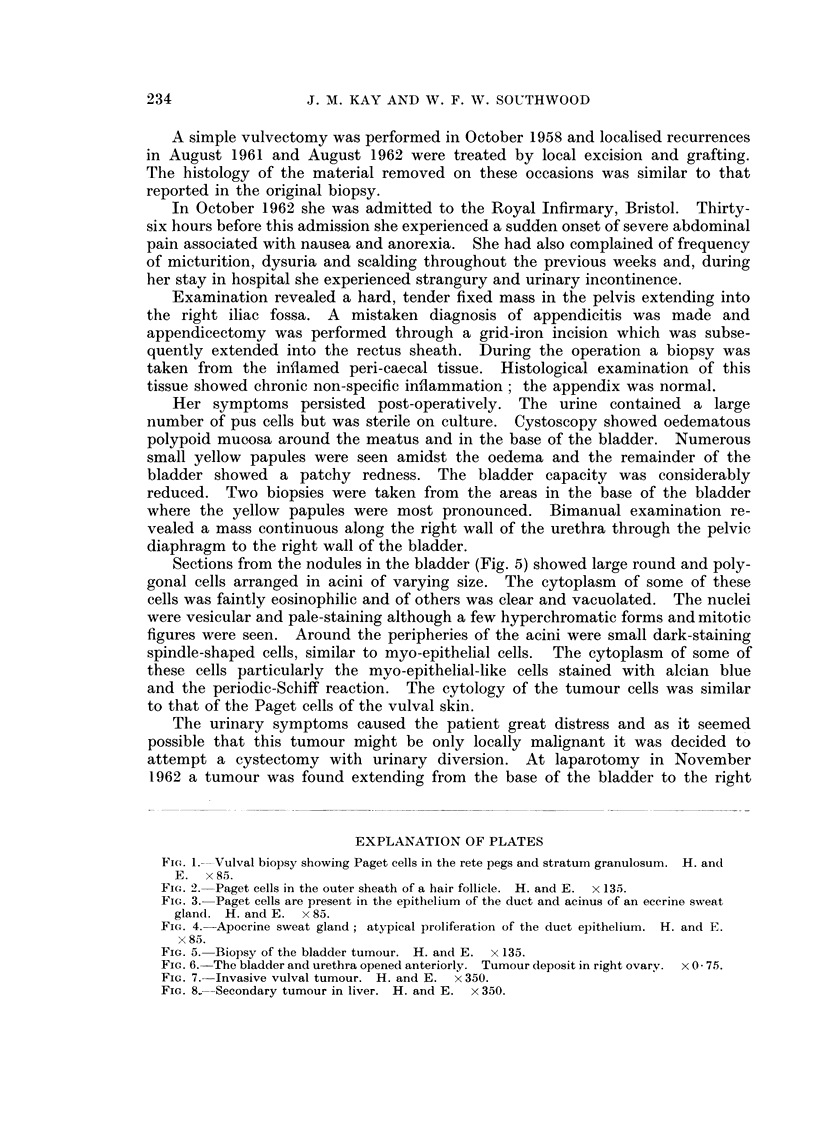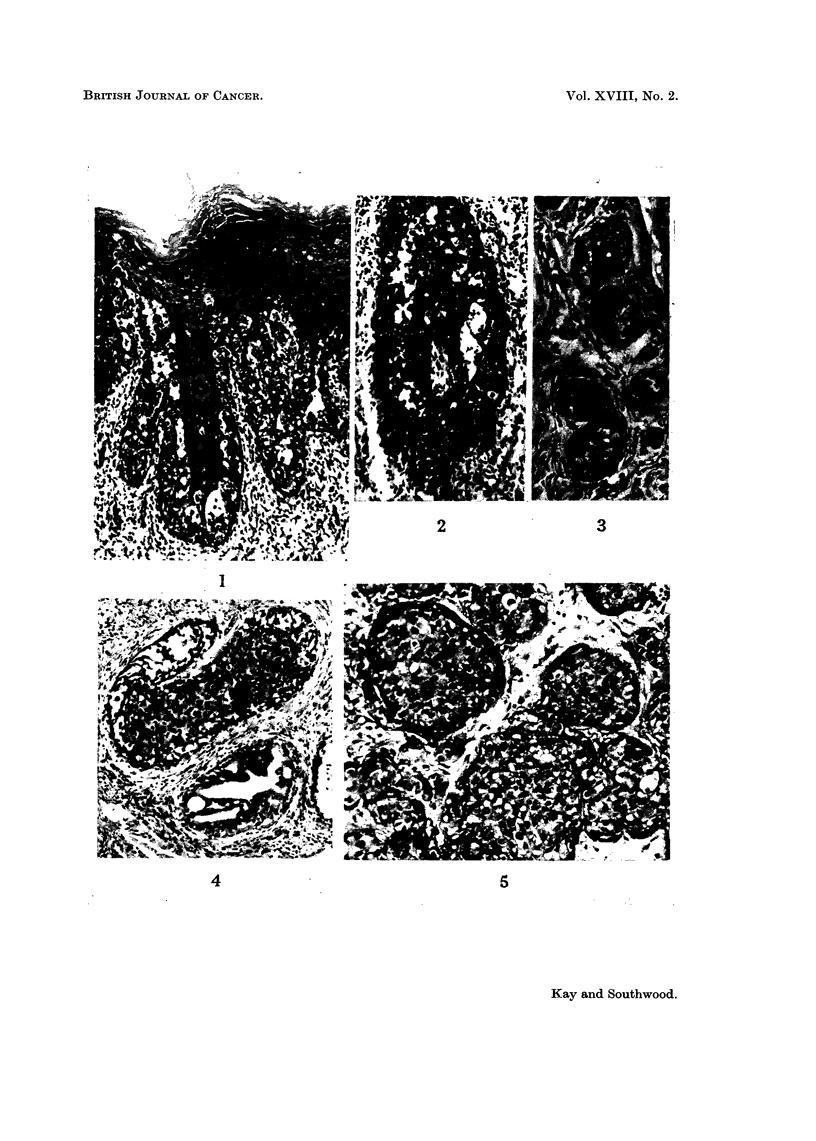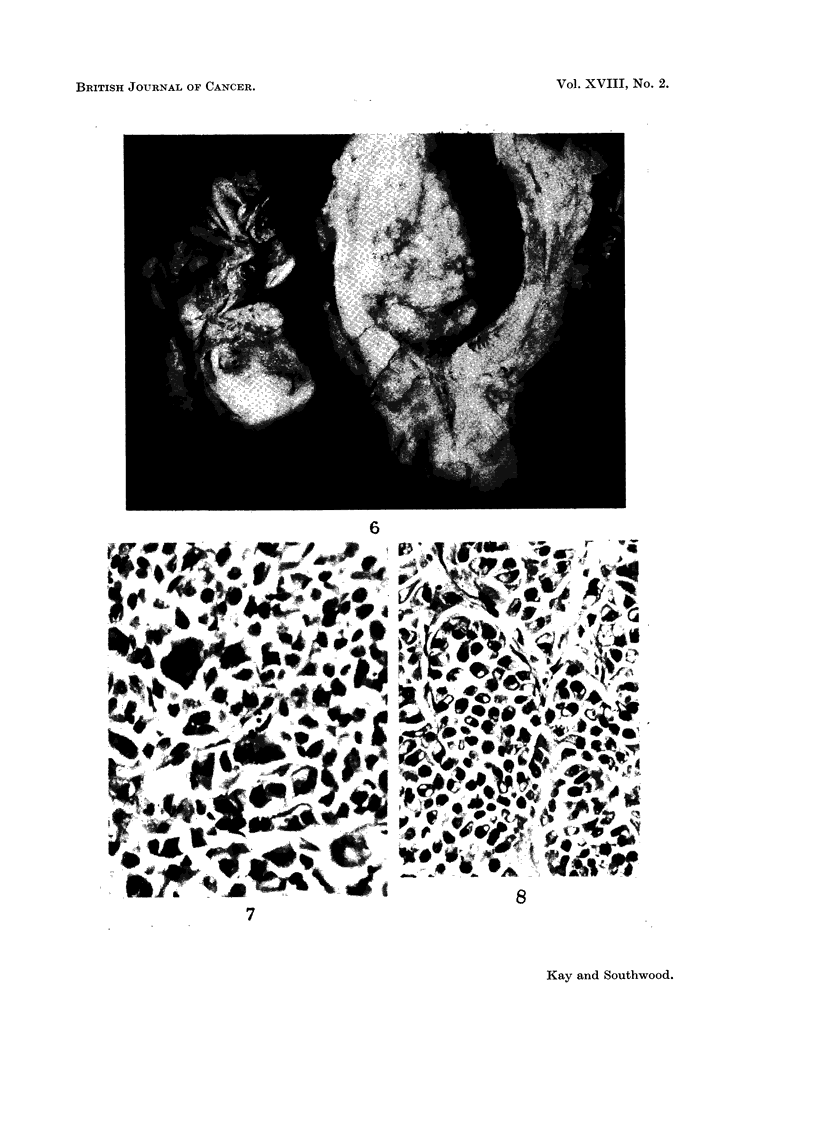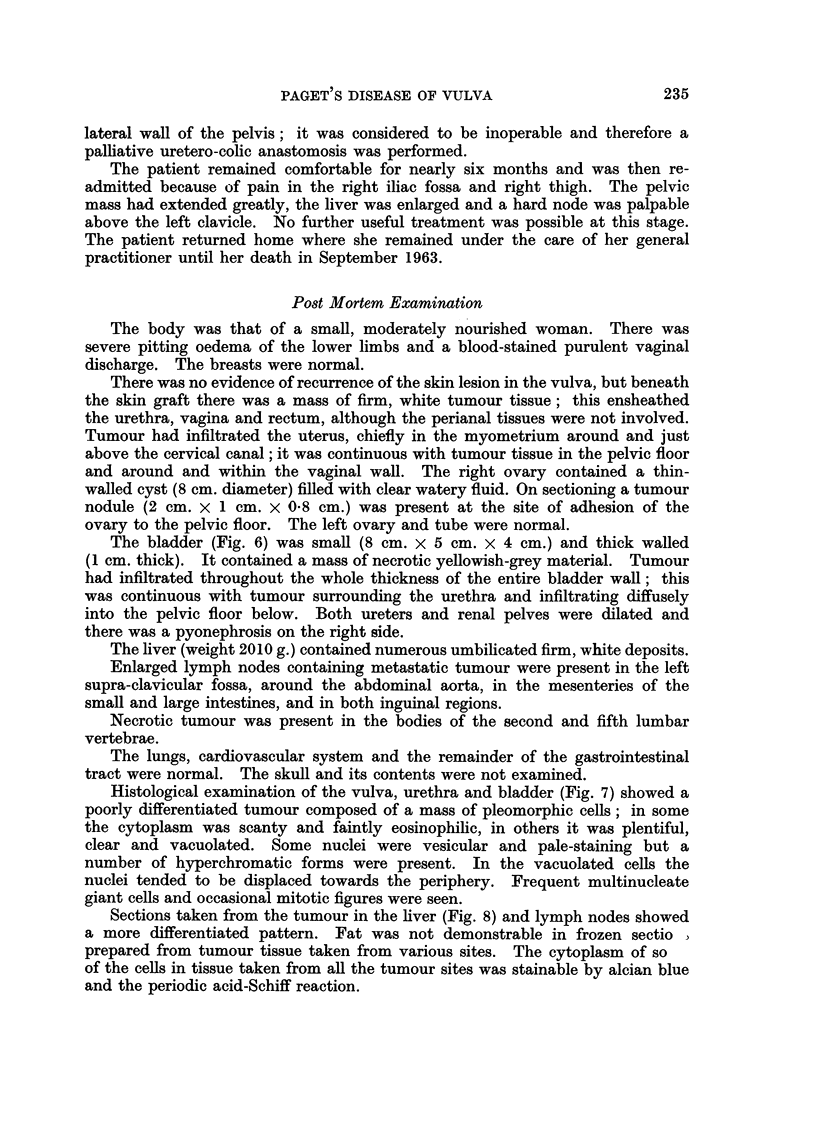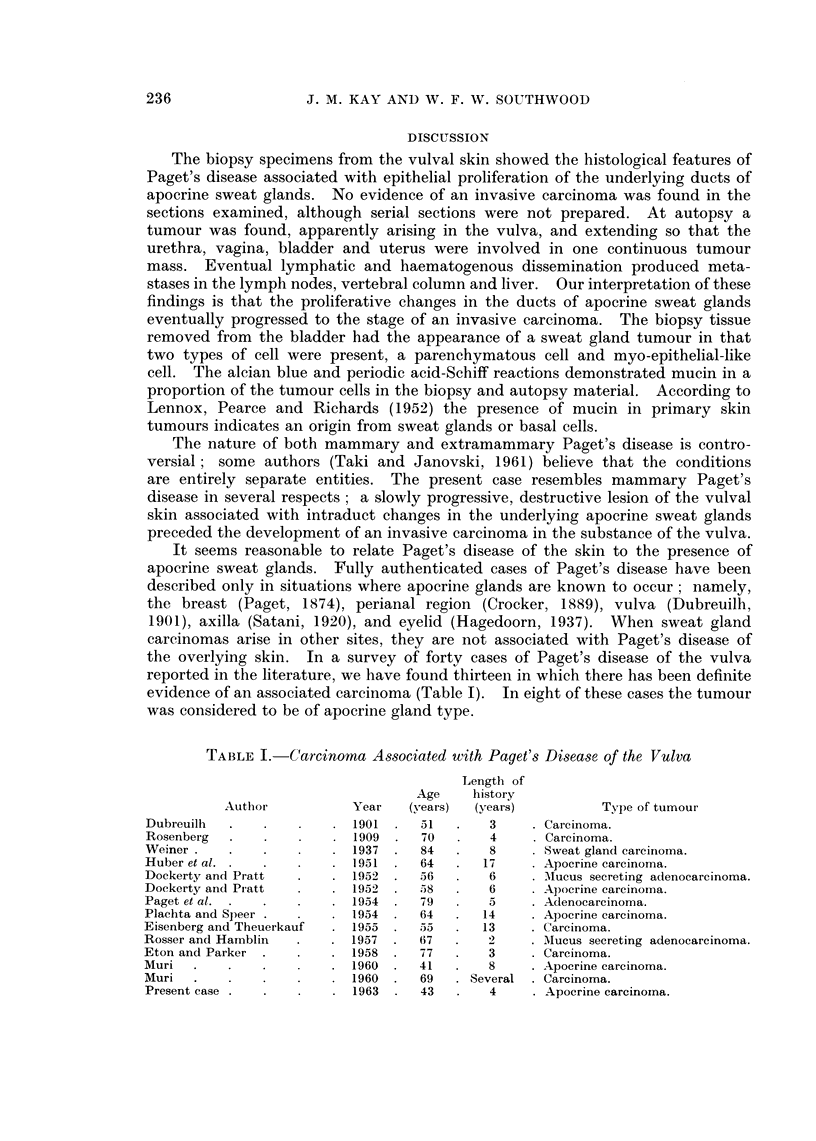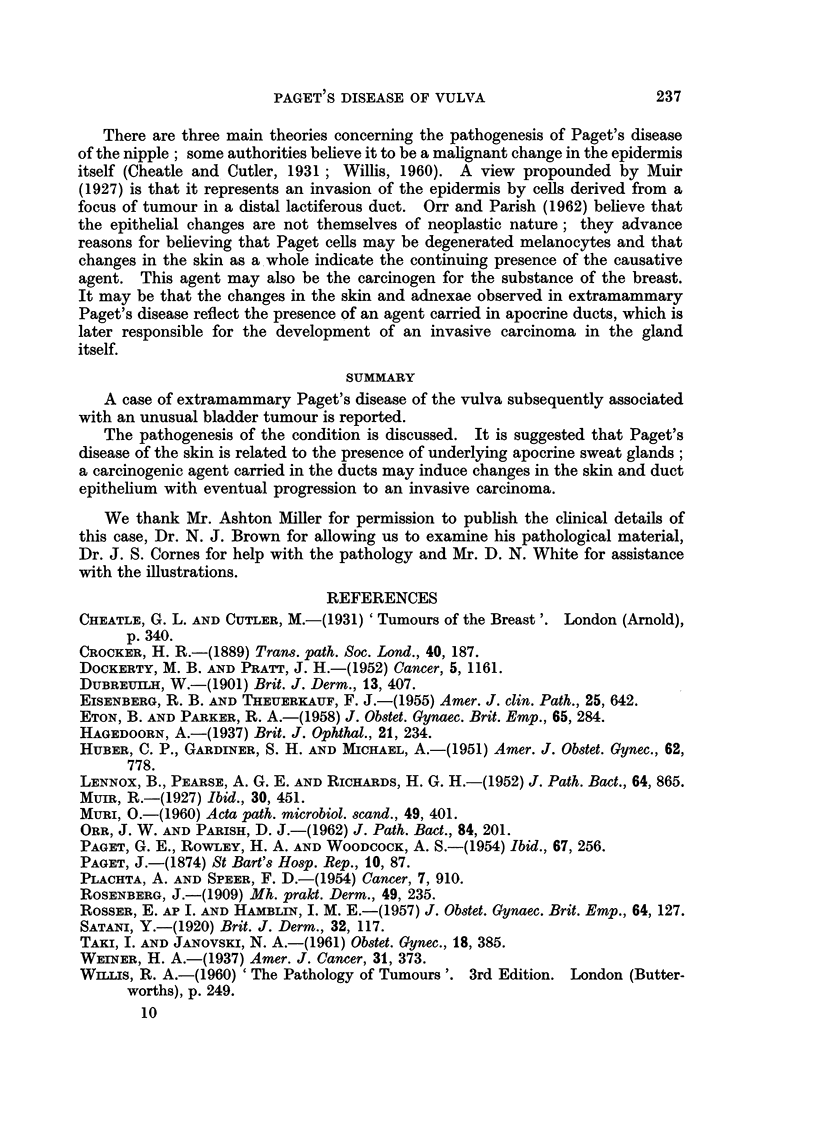# Paget's Disease of the Vulva Associated with an Unusual Bladder Tumour

**DOI:** 10.1038/bjc.1964.25

**Published:** 1964-06

**Authors:** J. M. Kay, W. F. W. Southwood

## Abstract

**Images:**


### PAGET'S DISEASE OF THE VULVA ASSOCIATED WITH AN UNUSUAL BLADDER TUMOUR

J. M. KAY* AND W. F. W. SOUTHWOOD

*From the Departments of Pathology and Surgery, University of Bristol*

Received for publication February 28, 1964

IN his classical description of disease of the mammary areola, Sir James Paget (1874) referred briefly to a similar condition affecting the glans penis and subsequently followed by a cancer in the substance of the organ. This reference has been the main justification for attaching the name extramammary Paget's disease to certain skin conditions occurring in other parts of the body. The naked eye and microscopic appearances of the mammary and extramammary lesions bear certain similarities. Their aetiology and pathogenesis remains highly controversial and it is not clear whether the two diseases bear any relation to each other despite a similarity in name.

In this paper we describe a patient who presented with extramammary Paget's disease of the vulva, and who later developed a most unusual bladder tumour.

### CASE REPORT

Miss N. M., an unmarried deaf mute, was 43 years old when she first attended Southmead Hospital, Bristol in 1958. She gave a two year history of pruritus vulvae which had not responded to local treatment, including hydrocortisone. Radiotherapy had not been used. On examination she was found to have an area of severe reddening with white patches involving both labia majora and minora, extending to the vaginal introitus. The inguinal nodes were not palpable.

A vulval biopsy was performed ; this showed hyperkeratosis and acanthosis of the epidermis (Fig. 1). The basal layer was disrupted and the rete pegs were distorted, elongated and distended by large clear Paget cells. These had vacuolated cytoplasm and contained vesicular nuclei situated either centrally or compressed to the periphery by the cytoplasm. The cells did not possess discernible prickles and were not keratinised. A small proportion contained hyperchromatic nuclei ; only a few mitotic figures were noted. Paget cells were seen to lie also in the outer sheaths of hair follicles (Fig. 2), and in the ducts and acini of eccrine sweat glands (Fig. 3). The epithelium of an apocrine sweat gland duct was abnormal (Fig. 4) ; the lumen was obliterated by a mass of atypical cells, some regular and pale-staining, others towards the periphery being large and vacuolated, resembling the Paget cells seen in the overlying epidermis. The dermis was oedematous and heavily infiltrated with a cellular exudate consisting of lymphocytes, mast cells and plasma cells. At no point were the Paget cells seen to have invaded the dermis. A material which stained with alcian blue and the periodic acid-Schiff reaction was present in some, but not all, of the Paget cells.

* Present address : Department of Pathology, University of Birmingham.

A simple vulvectomy was performed in October 1958 and localised recurrences in August 1961 and August 1962 were treated by local excision and grafting. The histology of the material removed on these occasions was similar to that reported in the original biopsy.

In October 1962 she was admitted to the Royal Infirmary, Bristol. Thirty-six hours before this admission she experienced a sudden onset of severe abdominal pain associated with nausea and anorexia. She had also complained of frequency of micturition, dysuria and scalding throughout the previous weeks and, during her stay in hospital she experienced strangury and urinary incontinence.

Examination revealed a hard, tender fixed mass in the pelvis extending into the right iliac fossa. A mistaken diagnosis of appendicitis was made and appendicectomy was performed through a grid-iron incision which was subsequently extended into the rectus sheath. During the operation a biopsy was taken from the inflamed peri-caecal tissue. Histological examination of this tissue showed chronic non-specific inflammation ; the appendix was normal.

Her symptoms persisted post-operatively. The urine contained a large number of pus cells but was sterile on culture. Cystoscopy showed oedematous polypoid mucosa around the meatus and in the base of the bladder. Numerous small yellow papules were seen amidst the oedema and the remainder of the bladder showed a patchy redness. The bladder capacity was considerably reduced. Two biopsies were taken from the areas in the base of the bladder where the yellow papules were most pronounced. Bimanual examination revealed a mass continuous along the right wall of the urethra through the pelvic diaphragm to the right wall of the bladder.

Sections from the nodules in the bladder (Fig. 5) showed large round and polygonal cells arranged in acini of varying size. The cytoplasm of some of these cells was faintly eosinophilic and of others was clear and vacuolated. The nuclei were vesicular and pale-staining although a few hyperchromatic forms and mitotic figures were seen. Around the peripheries of the acini were small dark-staining spindle-shaped cells, similar to myo-epithelial cells. The cytoplasm of some of these cells particularly the myo-epithelial-like cells stained with alcian blue and the periodic-Schiff reaction. The cytology of the tumour cells was similar to that of the Paget cells of the vulval skin.

The urinary symptoms caused the patient great distress and as it seemed possible that this tumour might be only locally malignant it was decided to attempt a cystectomy with urinary diversion. At laparotomy in November 1962 a tumour was found extending from the base of the bladder to the right

---

EXPLANATION OF PLATES

FIG. 1.—Vulval biopsy showing Paget cells in the rete pegs and stratum granulosum. H. and E. ×85.
FIG. 2.—Paget cells in the outer sheath of a hair follicle. H. and E. ×135.
FIG. 3.—Paget cells are present in the epithelium of the duct and acinus of an eccrine sweat gland. H. and E. ×85.
FIG. 4.—Apocrine sweat gland ; atypical proliferation of the duct epithelium. H. and E. ×85.
FIG. 5.—Biopsy of the bladder tumour. H. and E. ×135.
FIG. 6.—The bladder and urethra opened anteriorly. Tumour deposit in right ovary. ×0·75.
FIG. 7.—Invasive vulval tumour. H. and E. ×350.
FIG. 8.—Secondary tumour in liver. H. and E. ×350.

1
2
3
4
5

6

7

8

Kay and Southwood.

lateral wall of the pelvis; it was considered to be inoperable and therefore a palliative uretero-colic anastomosis was performed.

The patient remained comfortable for nearly six months and was then re-admitted because of pain in the right iliac fossa and right thigh. The pelvic mass had extended greatly, the liver was enlarged and a hard node was palpable above the left clavicle. No further useful treatment was possible at this stage. The patient returned home where she remained under the care of her general practitioner until her death in September 1963.

### *Post Mortem Examination*

The body was that of a small, moderately nourished woman. There was severe pitting oedema of the lower limbs and a blood-stained purulent vaginal discharge. The breasts were normal.

There was no evidence of recurrence of the skin lesion in the vulva, but beneath the skin graft there was a mass of firm, white tumour tissue; this ensheathed the urethra, vagina and rectum, although the perianal tissues were not involved. Tumour had infiltrated the uterus, chiefly in the myometrium around and just above the cervical canal; it was continuous with tumour tissue in the pelvic floor and around and within the vaginal wall. The right ovary contained a thin-walled cyst (8 cm. diameter) filled with clear watery fluid. On sectioning a tumour nodule (2 cm. × 1 cm. × 0·8 cm.) was present at the site of adhesion of the ovary to the pelvic floor. The left ovary and tube were normal.

The bladder (Fig. 6) was small (8 cm. × 5 cm. × 4 cm.) and thick walled (1 cm. thick). It contained a mass of necrotic yellowish-grey material. Tumour had infiltrated throughout the whole thickness of the entire bladder wall; this was continuous with tumour surrounding the urethra and infiltrating diffusely into the pelvic floor below. Both ureters and renal pelves were dilated and there was a pyonephrosis on the right side.

The liver (weight 2010 g.) contained numerous umbilicated firm, white deposits.

Enlarged lymph nodes containing metastatic tumour were present in the left supra-clavicular fossa, around the abdominal aorta, in the mesenteries of the small and large intestines, and in both inguinal regions.

Necrotic tumour was present in the bodies of the second and fifth lumbar vertebrae.

The lungs, cardiovascular system and the remainder of the gastrointestinal tract were normal. The skull and its contents were not examined.

Histological examination of the vulva, urethra and bladder (Fig. 7) showed a poorly differentiated tumour composed of a mass of pleomorphic cells; in some the cytoplasm was scanty and faintly eosinophilic, in others it was plentiful, clear and vacuolated. Some nuclei were vesicular and pale-staining but a number of hyperchromatic forms were present. In the vacuolated cells the nuclei tended to be displaced towards the periphery. Frequent multinucleate giant cells and occasional mitotic figures were seen.

Sections taken from the tumour in the liver (Fig. 8) and lymph nodes showed a more differentiated pattern. Fat was not demonstrable in frozen sectio[illegible] prepared from tumour tissue taken from various sites. The cytoplasm of so[illegible] of the cells in tissue taken from all the tumour sites was stainable by alcian blue and the periodic acid-Schiff reaction.

### DISCUSSION

The biopsy specimens from the vulval skin showed the histological features of Paget's disease associated with epithelial proliferation of the underlying ducts of apocrine sweat glands. No evidence of an invasive carcinoma was found in the sections examined, although serial sections were not prepared. At autopsy a tumour was found, apparently arising in the vulva, and extending so that the urethra, vagina, bladder and uterus were involved in one continuous tumour mass. Eventual lymphatic and haematogenous dissemination produced metastases in the lymph nodes, vertebral column and liver. Our interpretation of these findings is that the proliferative changes in the ducts of apocrine sweat glands eventually progressed to the stage of an invasive carcinoma. The biopsy tissue removed from the bladder had the appearance of a sweat gland tumour in that two types of cell were present, a parenchymatous cell and myo-epithelial-like cell. The alcian blue and periodic acid-Schiff reactions demonstrated mucin in a proportion of the tumour cells in the biopsy and autopsy material. According to Lennox, Pearce and Richards (1952) the presence of mucin in primary skin tumours indicates an origin from sweat glands or basal cells.

The nature of both mammary and extramammary Paget's disease is controversial ; some authors (Taki and Janovski, 1961) believe that the conditions are entirely separate entities. The present case resembles mammary Paget's disease in several respects ; a slowly progressive, destructive lesion of the vulval skin associated with intraduct changes in the underlying apocrine sweat glands preceded the development of an invasive carcinoma in the substance of the vulva.

It seems reasonable to relate Paget's disease of the skin to the presence of apocrine sweat glands. Fully authenticated cases of Paget's disease have been described only in situations where apocrine glands are known to occur ; namely, the breast (Paget, 1874), perianal region (Crocker, 1889), vulva (Dubreuilh, 1901), axilla (Satani, 1920), and eyelid (Hagedoorn, 1937). When sweat gland carcinomas arise in other sites, they are not associated with Paget's disease of the overlying skin. In a survey of forty cases of Paget's disease of the vulva reported in the literature, we have found thirteen in which there has been definite evidence of an associated carcinoma (Table I). In eight of these cases the tumour was considered to be of apocrine gland type.

TABLE I.—*Carcinoma Associated with Paget's Disease of the Vulva*

| Author | Year | Age (years) | Length of history (years) | Type of tumour |
|---|---|---|---|---|
| Dubreuilh | 1901 | 51 | 3 | Carcinoma. |
| Rosenberg | 1909 | 70 | 4 | Carcinoma. |
| Weiner | 1937 | 84 | 8 | Sweat gland carcinoma. |
| Huber *et al.* | 1951 | 64 | 17 | Apocrine carcinoma. |
| Dockerty and Pratt | 1952 | 56 | 6 | Mucus secreting adenocarcinoma. |
| Dockerty and Pratt | 1952 | 58 | 6 | Apocrine carcinoma. |
| Paget *et al.* | 1954 | 79 | 5 | Adenocarcinoma. |
| Plachta and Speer | 1954 | 64 | 14 | Apocrine carcinoma. |
| Eisenberg and Theuerkauf | 1955 | 55 | 13 | Carcinoma. |
| Rosser and Hamblin | 1957 | 67 | 2 | Mucus secreting adenocarcinoma. |
| Eton and Parker | 1958 | 77 | 3 | Carcinoma. |
| Muri | 1960 | 41 | 8 | Apocrine carcinoma. |
| Muri | 1960 | 69 | Several | Carcinoma. |
| Present case | 1963 | 43 | 4 | Apocrine carcinoma. |

There are three main theories concerning the pathogenesis of Paget's disease of the nipple ; some authorities believe it to be a malignant change in the epidermis itself (Cheatle and Cutler, 1931 ; Willis, 1960). A view propounded by Muir (1927) is that it represents an invasion of the epidermis by cells derived from a focus of tumour in a distal lactiferous duct. Orr and Parish (1962) believe that the epithelial changes are not themselves of neoplastic nature ; they advance reasons for believing that Paget cells may be degenerated melanocytes and that changes in the skin as a whole indicate the continuing presence of the causative agent. This agent may also be the carcinogen for the substance of the breast. It may be that the changes in the skin and adnexae observed in extramammary Paget's disease reflect the presence of an agent carried in apocrine ducts, which is later responsible for the development of an invasive carcinoma in the gland itself.

SUMMARY

A case of extramammary Paget's disease of the vulva subsequently associated with an unusual bladder tumour is reported.

The pathogenesis of the condition is discussed. It is suggested that Paget's disease of the skin is related to the presence of underlying apocrine sweat glands ; a carcinogenic agent carried in the ducts may induce changes in the skin and duct epithelium with eventual progression to an invasive carcinoma.

We thank Mr. Ashton Miller for permission to publish the clinical details of this case, Dr. N. J. Brown for allowing us to examine his pathological material, Dr. J. S. Cornes for help with the pathology and Mr. D. N. White for assistance with the illustrations.

### REFERENCES

CHEATLE, G. L. AND CUTLER, M.—(1931) ' Tumours of the Breast '. London (Arnold), p. 340.
CROCKER, H. R.—(1889) *Trans. path. Soc. Lond.*, **40**, 187.
DOCKERTY, M. B. AND PRATT, J. H.—(1952) *Cancer*, **5**, 1161.
DUBREUILH, W.—(1901) *Brit. J. Derm.*, **13**, 407.
EISENBERG, R. B. AND THEUERKAUF, F. J.—(1955) *Amer. J. clin. Path.*, **25**, 642.
ETON, B. AND PARKER, R. A.—(1958) *J. Obstet. Gynaec. Brit. Emp.*, **65**, 284.
HAGEDOORN, A.—(1937) *Brit. J. Ophthal.*, **21**, 234.
HUBER, C. P., GARDINER, S. H. AND MICHAEL, A.—(1951) *Amer. J. Obstet. Gynec.*, **62**, 778.
LENNOX, B., PEARSE, A. G. E. AND RICHARDS, H. G. H.—(1952) *J. Path. Bact.*, **64**, 865.
MUIR, R.—(1927) *Ibid.*, **30**, 451.
MURI, O.—(1960) *Acta path. microbiol. scand.*, **49**, 401.
ORR, J. W. AND PARISH, D. J.—(1962) *J. Path. Bact.*, **84**, 201.
PAGET, G. E., ROWLEY, H. A. AND WOODCOCK, A. S.—(1954) *Ibid.*, **67**, 256.
PAGET, J.—(1874) *St Bart's Hosp. Rep.*, **10**, 87.
PLACHTA, A. AND SPEER, F. D.—(1954) *Cancer*, **7**, 910.
ROSENBERG, J.—(1909) *Mh. prakt. Derm.*, **49**, 235.
ROSSER, E. AP I. AND HAMBLIN, I. M. E.—(1957) *J. Obstet. Gynaec. Brit. Emp.*, **64**, 127.
SATANI, Y.—(1920) *Brit. J. Derm.*, **32**, 117.
TAKI, I. AND JANOVSKI, N. A.—(1961) *Obstet. Gynec.*, **18**, 385.
WEINER, H. A.—(1937) *Amer. J. Cancer*, **31**, 373.
WILLIS, R. A.—(1960) ' The Pathology of Tumours '. 3rd Edition. London (Butterworths), p. 249.